# Clinical History of Patients with Hypertrophic Cardiomyopathy—How to Improve the Initiation Process of the Diagnosis?

**DOI:** 10.3390/jcm13175239

**Published:** 2024-09-04

**Authors:** Dominika Bieczek, Adrianna Ściślicka, Agnieszka Adamiec, Aleksandra Cader, Monika Wandasiewicz, Bartosz Basiaga, Małgorzata Niemiec, Katarzyna Mizia-Stec

**Affiliations:** 1 Students’ Scientific Society, First Department of Cardiology, Medical University of Silesia, 40-635 Katowice, Poland; ada.scislicka@gmail.com (A.Ś.); aagnieszka.adamiec@gmail.com (A.A.); aleksandra.cader156@gmail.com (A.C.); monikaa_1998@wp.pl (M.W.); bartoszbasiaga@gmail.com (B.B.); 2 Centre of the European Reference Network for Rare, Low Prevalence, or Complex Diseases of the Heart (ERN GUARD Heart), 1105 AZ Amsterdam, The Netherlands; kmiziastec@gmail.com; 3 First Department of Cardiology, Medical University of Silesia, 40-635 Katowice, Poland

**Keywords:** hypertrophic cardiomyopathy, heart failure, dyspnea, cardiomyopathy

## Abstract

**Background**: Regardless of genetic origin and recommended screening methods, hypertrophic cardiomyopathy (HCM) is commonly diagnosed late in the advanced stages of the disease. The aim of this study was to analyse the case history of patients with HCM in order to obtain an initiation of the diagnostic process. **Methods**: This study was a retrospective, tertiary, single-centre cohort analysis of 85 consecutive pts with HCM (mean age at the time of HCM diagnosis: 51 ± 15 years; F/M: 42/43) who were hospitalized during the period from 1 January 2013 to 31 December 2022. Type of referral to the hospital, the reason for hospitalization as well as accompanying symptoms, comorbidities, and family history were analysed to obtain an initiation of the diagnostic process. The analysis was limited to hospitalizations in which the diagnosis of HCM was stated for the first time. **Results**: An analysis of the type of referral to the hospital revealed the following data: 18% of patients were admitted as urgent hospitalizations (UHs) and 82% as elective hospitalizations (EHs). Among the UHs, the majority of patients were transferred from another hospital (13%), and among the EHs, 65% of patients were referred from a specialised outpatient medical care (SMC) facility. The majority of patients in both the UH and EH groups were symptomatic: 84% in the EH group (the most common symptom was exertional dyspnea in 56% of pts) and 93% in the UH group (the most common symptom was syncope in 60% of pts). Among the analysed population, the most frequent comorbidities were systemic hypertension (51%), lipid metabolism disorders (38%) and obesity (23%). **Conclusions**: A diagnosis of HCM is often made at an advanced age in symptomatic patients, mainly during an EH. Nearly one-fifth of the Polish HCM population is diagnosed during a UH after a sudden event, which suggests the need for improvements in medical care in Poland.

## 1. Introduction

Hypertrophic cardiomyopathy (HCM) is a relatively common genetic heart disease that is associated with a high risk of sudden cardiac death. The pathophysiology of HCM is complex, leading to significant variability in the morphological expression and natural course of the disease, which ranges from an asymptomatic course to heart failure [1]. The disease is caused by an increase in the wall thickness of the left ventricle, which results in a number of symptoms such as exertional dyspnea, angina, palpitations, vertigo and syncope. The incidence of sudden death is significantly increased in HCM, especially in young people affected by the disease [2]. Risk stratification in HCM should include a complete clinical cardiological assessment, which should also take into account new diagnostic features, e.g., cardiac magnetic resonance (CMR) imaging [3]. The goal of pharmacological treatment in HCM is to alleviate symptoms and includes pharmacotherapy and septum reduction therapies [4].

Preventive measures and active screening in the population should prevent incidents of sudden cardiac death. In Polish and European conditions, this disease entity is diagnosed relatively late [5]. Thanks to genetic screening, it is possible to identify people at risk of developing the disease who have not yet developed changes detectable by physical examination, e.g., LV hypertrophy [6]. The aim of this study was to analyse the preclinical history of adult HCM patients in order to obtain an initiation of the diagnostic process as well as to characterize the population with an early HCM diagnosis.

## 2. Materials and Methods

This study is a tertiary, single-centre, retrospective cohort analysis of 85 consecutive patients with HCM who were hospitalized in the 1st Department of Cardiology at the Leszek Giec Upper Silesian Medical Centre in Katowice, Poland; the Centre of the European Reference Network for Rare, Low-prevalence, or Complex Diseases of the Heart—ERN GUARD Heart. The analysis was limited to hospitalizations between 1 January 2013 and 31 December 2022 when the diagnosis of HCM was stated for the first time. Only the patients with HCM that were confirmed during the whole diagnostic process were included in the analysis.

The diagnosis was based on phenotypic characteristics of HCM confirmed using imaging tests including echocardiography and CMR. HCM was diagnosed when myocardial thickening was ≥15 mm in at least one left ventricular segment and could not be explained solely by an increased cardiac load. An obstructive form of HCM was diagnosed in patients with left ventricular outflow tract obstruction (LVOTO) with a gradient ≥30 mmHg at rest or after induction. The number of patients who underwent genetic testing, as well as information about detected genetic mutations associated with HCM, were not included in the analysis.

All data were obtained from the patients’ medical histories.

Overall, 85 (M/F: 43/42) patients with HCM were analysed. The mean age of the study group was 63.9 ± 12.9. The mean age at HCM diagnosis was 51 ± 15 years.

The following elements of the pts preclinical state were retrospectively analysed based on the medical history:

- type of referral to the hospital:
  - elective hospitalization (EH)—referrals made by first-line outpatient health care personnel (GP) or referrals from specialised outpatient medical care (SMC) personnel
  - urgent hospitalization (UH)—self-referral to the hospital emergency department, transfer from an ambulance to the hospital emergency department, or transfer of the patient from another facility for further diagnosis;
- accompanying signs and symptoms: systolic murmur, dyspnea at rest, dyspnea on exertion, angina pain, palpitations, syncope and vertigo;
- coexisting heart involvement at the moment of HCM diagnosis (in accordance with patients’ hospital and outpatient records): atrial fibrillation (AF)—any form of AF, supraventricular arrhythmias other than AF, ventricular arrhythmias and heart failure;
- comorbidities (in accordance with patients’ hospital and outpatient records): hypertension, diabetes, prediabetes, lipid metabolism disorders, coronary artery disease, obesity, chronic obstructive pulmonary disease (COPD), chronic kidney disease (CKD) (eGFR < 60 mL/min/m^2^) and gout;
- family history (in accordance with patients’ hospital and outpatient records): family history of HCM and sudden cardiac death in the family members.

Statistical analysis was performed using StatSoft Statistica version 13 software (StatSoft (Europe) GmbH, Hermannstr. 13, 20095 Hamburg, Germany). The analysis was conducted both for the entire group population and for subpopulations distinguished on the basis of hospital admission criteria—elective and urgent. Continuous variables were presented as means with standard deviation (SD). Meanwhile, categorical variables were presented as numbers and percentages. The Welch’s t-test or U Mann-Whitney test was used to compare quantitative data depending on distribution and variance homogeneity. Categorical data were compared using the Chi-square test with Yates or maximum likelihood correction. A *p*-value of less than 0.05 was considered as statistically significant.

## 3. Results

### 3.1. Type of Referral to the Hospital

**Figure 1:**
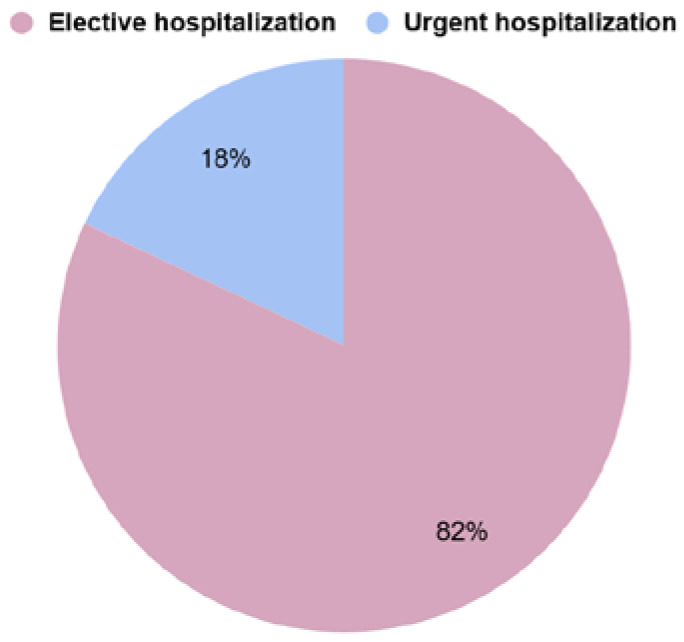
Percentage distribution of hospitalization types.

The types of referrals of patients to the hospital were analysed; EH applied to 68 (82%) patients and UH to 15 (18%) patients. The percentage distribution of hospitalization types is shown in Figure 1. 

**Figure 2:**
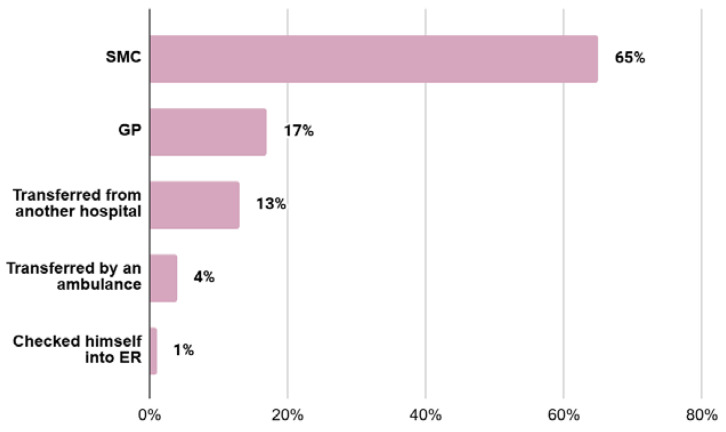
Percentage distribution of types of hospitalizations. SMC—specialised outpatient medical care; GP—first-line outpatient health care, ER—emergency room.

In the EH group, 14 (17%) people were referred by a GP and 54 (65%) patients were referred by SMC personnel. In the UH group, 1 (1%) patient checked himself into the emergency room, 3 (4%) people were transported by ambulance and 11 (13%) patients were transferred for diagnosis from another hospital (Figure 2). 

### 3.2. Signs and Symptoms at the Moment of HCM Diagnosis

**Figure 3:**
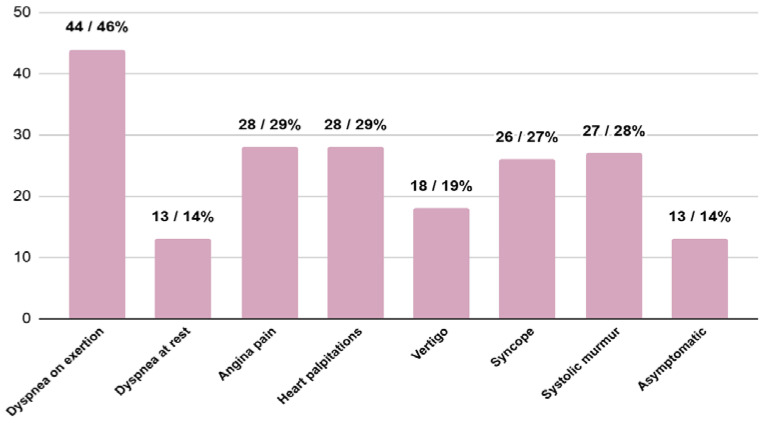
The number and percentage of patients with specific symptoms at the moment of diagnosis of HCM.

In the analysed group, 13 (15%) patients showed no symptoms, while 72 (85%) patients reported having symptoms at the time of diagnosis. The most frequently observed symptoms were dyspnea on exertion, angina pain and heart palpitations (Figure 3).

### 3.3. Coexisting Heart Involvement at the Moment of HCM Diagnosis

**Figure 4:**
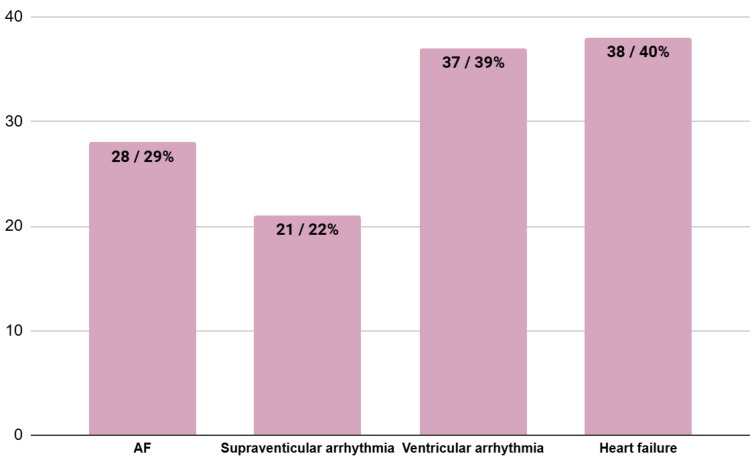
The number of patients with coexisting heart involvement at the moment of diagnosis of HCM. AF—atrial fibrillation.

The results of the analysis of coexisting heart involvement at the moment of HCM diagnosis are shown in Figure 4. The most common disorders were heart failure and ventricular arrhythmia. 

### 3.4. Comorbidities at the Moment of HCM Diagnosis

**Figure 5:**
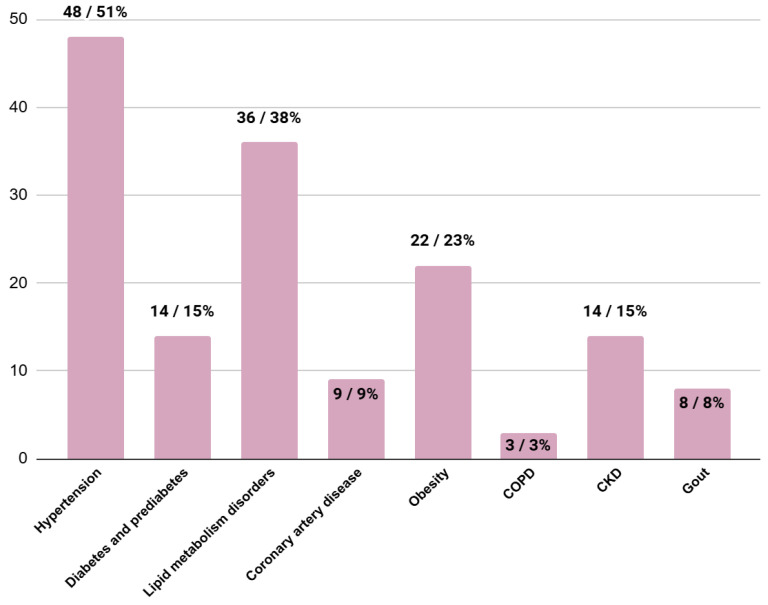
The number and percentage of patients with specific comorbidities. COPD—chronic obstructive pulmonary disease; CKD—chronic kidney disease.

The results of the analysis of comorbidities present at the time of diagnosis of hypertrophic cardiomyopathy are as follows: the most common disorders were hypertension, lipid metabolism disorders and obesity (Figure 5).

### 3.5. Family History at the Moment of HCM Diagnosis

**Figure 6:**
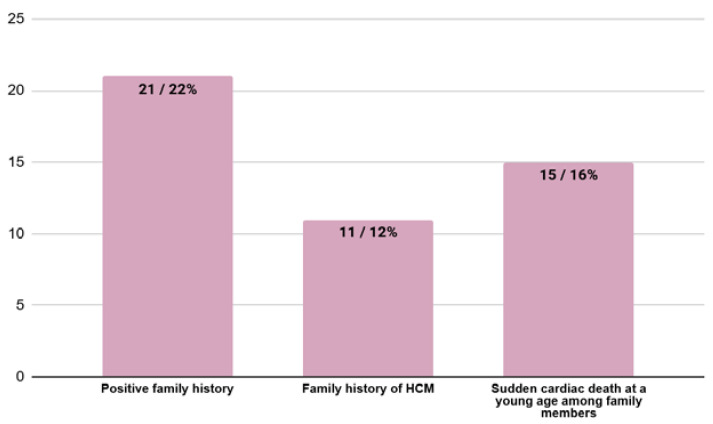
The number and percentage of patients with a positive family history. HCM—hypertrophic cardiomyopathy.

A total of 21 (22%) patients had a positive family history of either HCM or sudden cardiac death or both. A total of 11 (13%) patients had a family history of HCM, and 15 (18%) patients had family members who died due to sudden cardiac death (Figure 6). 

A statistically significant difference was found between the occurrence of HCM in the family and HCM diagnosis at a lower age in a patient (*p* = 0.04). 

### 3.6. Presence of LVOTO at the Moment of HCM Diagnosis

Data on the presence or absence of LVOTO were available for 62 patients. Within this subpopulation, the LVOTO was confirmed in 32 (52%) patients.

The percentage of symptomatic patients in the LVOTO (+) (81%) and LVOTO (−) (83%) subgroups was similar. A total of 30 (94%) patients with LVOTO were referred to the hospital in the elective way, compared to 21 (70%) patients in the LVOTO (−) subgroup (*p* = 0.01). 

### 3.7. Subgroup Characteristics and Analysis—EHs vs. UHs

The EH group consisted of 68 (82% of the general group) patients. The UH group consisted of 15 (18% of the general group) patients.

In the EH group, 11 (16%) patients were asymptomatic, while 57 (84%) had symptoms at the time of HCM diagnosis. The most common symptoms were dyspnea on exertion, heart palpitations and angina pain. In the UH group, 1 (7%) person was asymptomatic, and 14 (93%) patients presented symptoms of the disease at diagnosis. The most common symptoms were episodes of syncope, dyspnea on exertion, angina pain and systolic murmur upon physical examination. A statistically significant difference was present between the occurrence of the episodes of syncope (*p* = 0.01). 

Comparisons of the EH and UH groups revealed that diabetes, prediabetes and CAD were present statistically significantly more often in the EH group.

A total of 17 (25%) patients in the EH group and 4 (27%) patients in the UH group had a positive family history of either HCM or sudden cardiac death or both. A total of 7 (10%) patients in the EH group and 4 (27%) patients in the UH group had a family history of HCM. A total of 13 (19%) patients in the EH group and 2 (14%) patients in the UH group had a sudden cardiac death at a young age in the family.

Table 1 presents characteristics of each group. 

## 4. Discussion

Hypertrophic cardiomyopathy is diagnosed late. In this study, we wanted to verify the age of diagnosis and which symptoms of the disease accompanied it in our patient population. The aim was to determine the cause of hospitalization and to investigate why hospitalization was delayed in many cases, as the average age at diagnosis of HCM was ±51 years. According to our results, HCM is diagnosed in younger patients if they have a positive family history of HCM. Most often the patients were admitted for elective hospitalization. This study shows that the most common symptom of HCM is dyspnea on exertion, and only a small percentage of patients are asymptomatic. We also wanted to assess if the obstruction of the left ventricular outflow tract influenced the type of referral and symptoms at the moment of HCM diagnosis. 

In the study published in April 2024, the disease was described on the basis of 65,383 patients. The crucial finding is the high percentage of patients who were registered in the database with the CM code only once and the high percentage of patients who died after that first registration without returning to the health care system. On the other hand, our data clearly show that the first CM diagnosis was primarily established during a hospital stay [7,8]. In our study, we analysed the patients of only one reference centre where most of the patients were admitted as an elective hospitalization. The main symptom of the patients who were admitted as urgent hospitalizations was syncope. They are at greater risk of sudden cardiac arrest or recurrent syncope. Moreover, in our study, we analysed the clinical history of patients, and in the article, the results are based on the payer’s data.

Hypertrophic cardiomyopathy, once treated as a rare disease in young people, is now perceived as relatively common, with a statistical increase in diagnoses among middle-aged and older people. Moreover, based on the Sarcomeric Human Cardiomyopathy Registry, the mean age at diagnosis of HCM after 2010 was 51 ± 16 years, and this was confirmed in our study. It should be mentioned that in the cohort of the Sarcomeric Human Cardiomyopathy Registry, the women were older than the men [9].

Most patients report to cardiology specialists, family doctors or paediatricians. A visit to a family doctor or paediatrician is most often caused by the appearance of symptoms or incidental findings, e.g., abnormal electrocardiogram (ECG), audible heart murmur and abnormalities in imaging tests that were performed for other reasons (checkups at school, before taking part in sports competitions, before starting work or medical checkups); some patients are diagnosed during family screening after sudden cardiac death among relatives or due to diagnosis of HCM in the family [10,11,12,13]. The above observations are consistent with our results. About 80% of our patients reported to their GP or cardiologist as planned, from which the suspicion of HCM was mostly made by a specialist physician (in two-thirds of the patients). 

Only a small percentage of patients checked himself into the emergency room as UHs. These people appear mostly in secondary and tertiary care due to clinical heart failure, atrial or ventricular arrhythmia or suspected myocarditis [12]. About 10% of the patients included in our study were transferred to cardiology departments for diagnostics during hospitalization in other departments for other health reasons, and at the same time presented with cardiovascular symptoms. In the case of diagnostic problems and the need for an extended diagnostic process, patients are admitted to cardiology departments in order to be tested for HCM [8]. Some patients are diagnosed at tertiary health centres if they are screened because of a family history of HCM. Many patients take part in clinical trials, which also lead to the diagnosis of the disease among their relatives [9,14,15]. A positive correlation using the Chi-squared test was found between the occurrence of HCM in the family and HCM diagnosis at a lower age (*p* = 0.04).

Furthermore, HCM is a disease whose clinical assessment and management decisions are made in the outpatient setting. Other symptoms range from asymptomatic to heart failure or sudden cardiac death, which means patients need to be properly checked and diagnosed by a specialist. In our analysis, 14% of patients did not have any symptoms. Diagnoses may be difficult due to phenotypic heterogeneity. The first symptom is often a heart murmur, specifically a systolic ejection murmur, which is best heard between the apex and the left border of the sternum and occurs upon loading to reduce preload or afterload [16]. The assessment of a patient with HCM requires an interview that takes into account symptoms such as dyspnea on exertion, syncope, chest pain and then palpitations. In our study, the most common symptom was exertional dyspnea. The most important problem in HMC is a major differential diagnosis that may result in an under-recognized outcome or a delayed diagnosis. It is crucial to clarify the secondary consequences of cardiac death. Assessing the risk of cardiac death is of great importance because sudden death is the leading cause of death in patients with HCM [16]. A total of 16% of patients in our study group had a sudden cardiac death at a young age in the family.

A physical examination and the use of noninvasive tests including genetic tests are also an essential element. HCM may be inherited as an autosomal dominant disease associated with variants in genes encoding cardiac sarcomere proteins involved in contractile function [6,10,17]. If the same mutation is detected in one or more relatives, an initial clinical assessment is recommended, followed by repeated long-term assessments at intervals depending on the advancement of the disease or the occurrence of cardiovascular symptoms (ECG, echocardiography and CMR) [16]. 

Chest pain and clinical signs of myocardial ischemia in the absence of epicardial coronary artery disease (CAD) are common in patients with HCM and are mostly caused by small vessel disease [18]. Also in our follow-up, angina pain was one of the most common symptoms. Among the patients in the EH group, 34% of patients experienced angina pain. On the other hand, in the UH group, 33% of patients suffered from angina pain. Moreover, statistically significantly more patients in the UH group were diagnosed with CAD than patients in the EH group. This can be explained by the fact that CAD can cause worsening of the cardiac condition. Furthermore, according to our study, a risk factor of urgent referral is the occurrence of diabetes or prediabetes. It is well known that hyperglycemia is a risk factor for atherosclerosis and heart disease. Atrial fibrillation occurs in almost 1 in 5 patients and is associated with a significant risk of stroke [4,19]. In a study by López-Ponce de Leon JD et al., atrial fibrillation was the most common arrhythmia and was recorded in 22% of patients during the follow-up period [20], but in our study population, up to 29% had atrial fibrillation. A total of 32% of patients in the EH group and 40% of patients in the UH group had atrial fibrillation. Syncope and presyncope occur in 1 in 4 HCM patients, with mechanisms including supraventricular arrhythmia, sinus node dysfunction, complete heart block, ventricular arrhythmia, LVOTO, inappropriate vasodilation, volume depletion and diastolic dysfunction-mediated hypotension [4]. In HCM, syncope is reported in nearly 16% of patients [21]. In our review, 27% of patients had episodes of syncope. Moreover, almost 25% of patients in the EH group and 60% of patients in the UH group had experienced episodes of syncope. This difference was statistically significant (*p* = 0.01). This may mean that patients are more likely to ignore symptoms that they believe are not immediately life-threatening, while symptoms that cause them higher levels of anxiety and stress prompt them to seek medical assistance more quickly.

The occurrence of LVOTO had an influence on the symptoms and type of referral as well. Our study established that there was a statistically significant difference in the presence of systolic murmur on physical examination in the patients with LVOTO. This is confirmed in the literature as well [22]. Moreover, people with systolic murmur have a higher frequency of EH, so it may suggest that the presence of systolic murmur leads the GP or SMC personnel to suspect the presence of the disease earlier. 

The hypertrophic cardiac phenotype can result from a variety of causes, so precise differentiation of cardiomyopathy phenocopies is crucial for appropriate treatment. Echocardiography is the standard tool for diagnosing cardiac hypertrophy, but cardiac magnetic resonance imaging offers greater precision and reliability in diagnoses of the phenotype thanks to advanced tissue characterization. In particular, T1 and T2 mapping in CMR imaging improves the accuracy of diagnosis and can be used to evaluate the effectiveness of therapy in infiltrating diseases. Additionally, the presence of contrast enhancement in CMR imaging is an important prognostic factor in the evaluation of HCM [23].

The limitations of the study are as follows: we only had access to patients’ medical records from the time of HCM diagnosis; the patients were not re-examined; and the patients’ interviews were not collected. Information about comorbidities and complications present at the time of diagnosis of HCM also came only from patients’ medical records. Moreover, a diagnosis of HCM was made based on symptoms, and no genetic tests were performed in patients to confirm an HCM diagnosis. A major drawback is the small sample size. Moreover, we have no data on LVOTO in all patients in the study. These limitations could impact the generalizability of our findings and should be considered when interpreting the results.

This work stands out from other research studies on this topic in several important respects. First, this is an analysis of consecutive patients of 1st Department of Cardiology at the Leszek Giec Upper Silesian Medical Centre in Katowice, Poland; the Centre of the European Reference Network for Rare, Low-prevalence, or Complex Diseases of the Heart - ERN GUARD Heart hospitalized between 1 January 2013 and 31 December 2022, which includes long-term follow-ups and ensures accurate data from a single centre. This work focuses on a detailed analysis of patients’ medical history, with an emphasis on the moment of initiation of the diagnostic process. This approach allows us to understand which factors contribute to delays in the diagnosis of HCM. Additionally, this work presents a detailed analysis of patients’ symptoms at the time of diagnosis and an analysis of coexisting diseases and family burdens.

## 5. Conclusions

It should be noted that a diagnosis of HCM is often made at an advanced age in symptomatic patients mainly during an EH. Nearly one-fifth of the Polish HCM population is diagnosed during a UH after a sudden event, which suggests the need for improvements in medical care in Poland. An early diagnosis and prompt care are crucial to prevent as many possible complications of the disease as possible. 

## Figures and Tables

**Table 1:** Characteristics of the general group and each subgroup depending on the type of admission to the hospital (EH vs UH) at the time of HCM diagnosis.

General Characteristic	General Group N = 85	Elective N = 68	Urgent N = 15	*p*
Age (years ± SD)	51 ± 15.2	52 ± 14.0	46 ± 19.5	0.36
Sex male (n/%) female (n/%)	43/45% 42/44%	34/41% 34/41%	8/10% 7/8%	0.82
Asymptomatic (n/%)	13/14%	11/16%	1/7%	0.34
Signs and symptoms (n/%)	71/76%	57/84%	14/93%	0.34
Systolic murmur (n/%)	27/28%	22/32%	5/33%	0.97
Dyspnea at rest (n/%)	13/14%	11/24%	2/13%	0.78
Dyspnea on exertion (n/%)	44/46%	38/56%	6/40%	0.26
Angina pain (n/%)	28/29%	23/34%	5/33%	0.97
Heart palpitations (n/%)	28/29%	25/37%	3/20%	0.21
Episodes of syncope (n/%)	26/27%	17/25%	9/60%	**0.01**
Vertigo (n/%)	18/19%	15/22%	3/20%	0.86
Coexisting heart involvement				
Atrial fibrillation (n/%)	28/29%	22/32%	6/40%	0.57
Supraventricular arrhythmias other than atrial fibrillation (n/%)	21/22%	18/27%	3/20%	0.60
Ventricular arrhythmias (n/%)	37/39%	28/41%	8/53%	0.39
Heart failure (n/%)	38/40%	33/49%	4/27%	0.11
Comorbidities				
Hypertension (n/%)	48/51%	41/60%	7/47%	0.33
Diabetes and prediabetes (n/%)	14/15%	9/13%	5/33%	**0.02**
Lipid metabolism disorders (n/%)	36/38%	31/46%	4/27%	0.34
Coronary artery disease (n/%)	9/9%	4/6%	4/27%	**0.01**
Obesity (n/%)	22/23%	18/27%	3/20%	0.54
COPD (n/%)	3/3%	2/3%	1/7%	0.40
CKD (n/%)	14/15%	10/15%	3/20%	0.43
Gout (n/%)	8/8%	6/9%	1/7%	0.91
Family history				
Positive family history (HCM in family, sudden cardiac death in family or both) (n/%)	21/22%	17/25%	4/27%	0.89
HCM in family (n/%)	11/12%	7/10%	4/27%	0.09
Sudden cardiac death in family (n/%)	15/16%	13/19%	2/13%	0.60

SD—standard deviation; COPD—chronic obstructive pulmonary disease; CKD—chronic kidney disease; HCM—hypertrophic cardiomyopathy. The bold means that the result is statistically significant.

## Data Availability

The datasets presented in this article are not readily available because of patient privacy and legal/ethical issues.
